# Application of *Polypodiopsida* Class in Nanotechnology–Potential towards Development of More Effective Bioactive Solutions

**DOI:** 10.3390/antiox10050748

**Published:** 2021-05-08

**Authors:** Irina Fierascu, Radu Claudiu Fierascu, Camelia Ungureanu, Oana Alexandra Draghiceanu, Liliana Cristina Soare

**Affiliations:** 1 National Institute for Research & Development in Chemistry and Petrochemistry-ICECHIM, 060021 Bucharest, Romania; irina.fierascu@icechim.ro; 2 Faculty of Horticulture, University of Agronomic Sciences and Veterinary Medicine of Bucharest, 011464 Bucharest, Romania; 3 Department of Science and Engineering of Oxide Materials and Nanomaterials, University “Politehnica” of Bucharest, 011061 Bucharest, Romania; 4 Department of General Chemistry, University “Politehnica” of Bucharest, 011061 Bucharest, Romania; 5 Department of Natural Sciences, University of Pitesti, 1 Targu din Vale Str., 110040 Pitesti, Romania; oana.draghiceanu@upit.ro (O.A.D.); cristina.soare@upit.ro (L.C.S.)

**Keywords:** phytosynthesis, antioxidant, antimicrobial, *Polypodiopsida*, nanotechnology

## Abstract

The area of phytosynthesized nanomaterials is rapidly developing, with numerous studies being published yearly. The use of plant extracts is an alternative method to reduce the toxic potential of the nanomaterials and the interest in obtaining phytosynthesized nanoparticles is usually directed towards accessible and common plant species, ferns not being explored to their real potential in this field. The developed nanoparticles could benefit from their superior antimicrobial and antioxidant properties (compared with the nanoparticles obtained by other routes), thus proposing an important alternative against health care-associated and drug-resistant infections, as well as in other types of applications. The present review aims to summarize the explored application of ferns in nanotechnology and related areas, as well as the current bottlenecks and future perspectives, as emerging from the literature data.

## 1. Introduction

The term “nano” is encountered in all aspects of our daily life, often being regarded as the ultimate “bottleneck” breaker in various technological areas [1,2,3]. Day by day, nanomaterials represent a more and more a common aspect of our lives, in as much as we are becoming accustomed to nanomaterials-based personal care products, pharmaceutical products or even agricultural products [4]. The “nano” approach can help to improve the solubility of poorly water-soluble active substances and increase their bioavailability [5], increase storage stability of active substances, and develop more efficient drug carriers, thus leading to next generation nanomedicine tools [6]. In this “nano-rush” we often forget that the anthropogenically engineered nanoparticles raise several questions regarding their safe use. Although the use of nanomaterials should not be considered as intrinsically harmful [5], their negative potential should be evaluated on the entire production chain, from their synthesis to final application and disposal [7], different review works describe toxicological aspects related to the use of such nanomaterials [7,8].

Considering their biomedical applications, as well as their behavior in relevant environments, the nanomaterials can be divided into disintegrating and non-disintegrating nanomaterials. The disintegrating nanomaterials are usually represented by composites, in which the encapsulating carrier structure disintegrates in order to allow the release of the active substance [9,10,11], while the non-disintegrating nanomaterials are represented by nanoparticles (usually metallic nanoparticles) applied for imaging techniques or even as active materials [12,13]. The literature provides several reviews regarding the application of nanoparticles in medicine [14,15,16], and as such any further discussion would be beyond the aim of the current review.

Consideration of alternative methods to reduce the toxic potential of the nanomaterials led to the proposal of new synthesis approaches, respecting the principles of “green chemistry”, among which the phytosynthesis (obtaining nanomaterials and nanoparticles using natural extracts) represents one of the most important routes [17]. These types of nanomaterials present lower risks related to their applications [18], compared with similar nanomaterials obtained via classical chemical routes, and, in some cases, enhanced properties [17,19]. As such, the scientific studies regarding the phytosynthesis of nanomaterials grew exponentially over the last decade [18,19]. The phytosynthesis of metallic nanoparticles represents a very complex process, involving the reduction in the metallic precursor, followed by their capping by different phytoconstituents, specific for each plant [17,18]. The reduction in stabilization of metal ions is achieved by the synergic action of biomolecules (such as proteins, organic acids, amino acids, or vitamins), and secondary metabolites (including polyphenols, flavonoids, terpenoids, alkaloids, etc.) [20,21].

Appearing in the Devonian period [22], the ferns represent a “silent witness” of the earth’s transformation, some species remaining unchanged for over 180 million years [23]. As the human civilization grew in the presence of this class of plants, their potential uses were explored from its early beginning, being used as a food source, or in folk medicine [24,25]. Although in our days are considered of minor economic importance, mainly regarded as ornamental plants, ferns can provide important information regarding their metabolites and biological properties [26]. Despite this “rediscovery” of ferns, their application in nanotechnology does not represent a main focus of the scientific literature, which is hardly explainable, considering how widespread they are.

**Figure 1:**
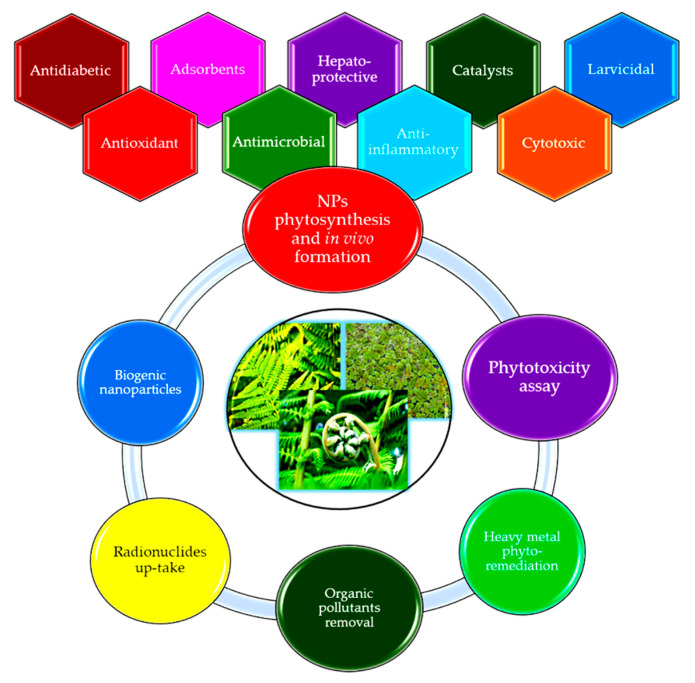
*Polypodiopsida* species: applications in nanotechnology and related areas to be discussed in the following chapters.

The interest in obtaining phytosynthesized nanoparticles (NPs) is usually directed to accessible and common plants extracts or plants waste extracts [27], ferns not being explored to their real potential in this field. A graphical summary of the applications of *Polypodiopsida* species in the field of nanomaterials and other related area that will be discussed in the present work is presented in Figure 1. The following chapters will present the phytosynthesis of metal and metal oxides nanoparticles using different plants from the *Polypodiopsida* class, their potential applications as antioxidant, antimicrobial, anti-inflammatory, cytotoxic, antidiabetic, hepatoprotective, or larvicidal agents, as well as their use as adsorbents or catalysts. The potential application of these species for phytotoxicity assays, phyto-remediation, organic pollutants and radionuclides up-take, as well as for obtaining biogenic nanoparticles, are discussed. The main components involved in the phytosynthesis process are presented, when identified by the cited authors.

## 2. The Polypodiopsida Class

Appearing in the *Palaeozoic* era, ferns belong to the group of vascular plants or tracheophytes, plants that have conductive xylem and phloem tissues, necessary for the transport of water and nutrients [28].

Based on recent phylogenetic data, the *Pteridophyte Phylogeny Group* [29] classified current species of the class *Polypodiopsida* (Table 1) into four subclasses, 11 orders, 48 families, 319 genera, and an estimated 10,578 species.

The subclass *Equisetidae* includes a single current genus, *Equisetum*, with species widespread in humid habitats, characterized by the presence of an underground rhizome-type stem from which aerial ridged stems are formed. The determination of the sex of the potential bisexual gametophyte is influenced by environmental factors [30], a phenomenon known as environmental sex determination (ESD) [31]. Some species of *Equisetum* are medicinal [32].

*Ohioglossidae* are ferns that form from the rhizome (usually) a single leaf, not-circinate in the bud, a leaf that has a sterile, assimilating and a fertile segment on which sporangia are formed. *Ophioglossidae* also include *Psilotales* which are perennials, with rootless rhizome, and green, photosynthetic aerial stems.

The subclass *Marattiidae* includes ferns found in tropical forests. They have an aerial stem terminated with large leaves, with sporangia growing in the synangia or grouped in sori. Some species are ornamental or food [32].

As can be seen from Table 1, most fern species belong to the *Polypodiidae* subclass. They are plants widespread in all climatic zones, but most species are found in tropical mountain areas [33]. They are less common in arid and semi-arid areas [34], salt marshes, mangrove swamps, etc. [30]. Being adapted to different environments, ferns have life forms similar to spermatophyta [30]. Thus, tree species (phanerophyte, *Cyathea*), lianas (*Lygodium*) and epiphytes (*Platycerium*) are found in tropical areas, hemicryptophytes with perennating buds at the soil surface, and have a predominant life form in subhumid temperate ecosystems (*Dryopteris*) [35]. In dry environments, the most encountered form is chamaephytic [35]. There are also geophyte species (*Pteridium*), as well as annual species (therophyte, *Anogramma*). The order *Salviniales* includes hydrophytes, adapted to the aquatic environment.

In areas with high biodiversity, ferns have been used for millennia both in traditional medicine and as food plants, being consumed after heat processing, drying, etc. [36].

The *sporophyte* is represented by the plant itself, consisting of the root, stem and leaves with sporangia. The roots are adventitious, of endogenous origin, as the embryonic root disappears early. From a functional point of view, the roots fix the ferns in the soil and absorb water and mineral salts. Most species have rhizome-type underground stems, but there are also above-ground stems in tree species or aquatic stems. The stems support the leaves and transport the sap through a wide range of steles [37].

The leaves are large, megaphylls [38] and are the dominant part of the plant in most species [39]. The leaf blade can be whole (*Asplenium scolopendrium*) or variously incised.

From a structural point of view, the leaves of evolved ferns have an anatomy similar to that of spermatophytes [30]. From a functional point of view, the leaves have a role in photosynthesis, respiration, transpiration, but also in the formation and protection of sporangia. The fertile leaves, producing sporangia, can have the same shape and size as the sterile ones (monomorphic leaves), or they can be different (dimorphic leaves) [39]. The leaf buds are circinately coiled (fiddleheads) and present aerophores [39,40].

Sporangia are multicellular, grouped in *sori* (in most species) or in *sporocarps* (in aquatic ferns).

In most *Polypodiidae*, the spores are isospores, morphologically identical, undifferentiated sexually; less often they are heterospores (small spores-microspores, formed in microsporangia and large-macrospores, formed in macrosporangia) (*Salviniales*: *Salvinia, Marsilea,* etc.).

Spores released from sporangia germinate and thus form a *gametophyte* (often called *prothalli*) [37] of various shapes.

The morphology of the gametophyte is diverse, as a result of adapting to very different habitats in which ferns are encountered, which has led to the emergence of different strategies for survival [41]. Thus, the heart-shaped gametophyte is annual, common to most terrestrial species. The strap shaped gametophyte is perennial, found in epiphytic species of *Polypodiaceae* and *Elaphoglossaceae*. The perennial, ribbon-like gametophyte is characteristic of some *Hymenophyllaceae*. The last two categories produce *gemmae* that are dispersed by wind, water or other factors and generate new gametophytes.

From the germination of isospores results *protals*, on which are formed both the antheridia, in which they differentiate the mobile male gametes—*antherozoids*, and the *archegonia*, in which the female gamete—the *egg cell* is differentiated. From the germination of heterospores, microprotals with antheridia and macroprotals with archaegonia are formed. The above-ground gametophyte is fixed in the soil by rhizoids, and gametangia are formed on its underside. Sex determination of the gametophyte is performed by antheridiogens [42] which influence the formation of anteridia and fertilization [43] having an important role in increasing the genetic variability of fern populations [44].

As already mentioned, the phytosynthesis mechanism (previously presented by our group in several works [17]) assigns a major role to the synergic action of multiple biomolecules and secondary metabolites. As such, the chemical composition of the ferns further used in nanotechnology represents an important aspect to be considered, for a successful phytosynthesis. The particular phytocomponents and their concentration strongly varies with each species, plant part (rhizome, fronds, petiole, etc.), as well as with environmental factors (as is the case for any other plant types). Secondary metabolites of the terpenoid class (diterpenoids, triterpenoids, sesquiterpenoids, etc.), the phenolic group (phenylpropanoid derivatives and others), the flavonoid class, and the alkaloid class are bioactive compounds that have been identified in ferns and give them numerous therapeutic properties: antioxidants, antimicrobial, antimalarial, anti-inflammatory, diuretic, cytotoxic, antitumor, etc. [26].

The most encountered secondary metabolites are the flavonoid glycosides and flavonols (such as kaempferol, quercetin, luteolin and apigenin derivatives, quercetin, quercitrin, hyperoside, etc.), xanthones, spiropyranosyl derivatives and triterpenoids [25,45]. Other flavonoids with various bioactivity, such as apigenin, luteolin, naringenin, kaempferol, violanthin or isoviolanthin, were identified in ferns [26,46].

The total polyphenols content (TPC), a parameter often associated with the nanoparticle phytosynthesis (a higher TPC value leading to smaller nanoparticles dimensions [47]) varies within different families and species of ferns, possible due to the different environmental factors that characterize their habitats. Thus, *Dryopteris affinis* leaves were found to contain 887 mg GAE (gallic acid equivalent)/100g fresh weight (f.w.), while in *D. filix-mas* the total polyphenols content was found to be 2340 mg GAE/100 g f.w. [48]. Usually, the TPC correlates positively with antioxidant activity, both for the extracts and the resulting NPs [47]. Thus, in the species *Dryopteris affinis* ORAC (oxygen radical absorbance capacity) assay antioxidant activity was found to be 128.18 µmol Trolox equivalents (TE)/g f.w., while in *D. filix-mas* it was recorded as 421.90 µmol TE/g f.w. [48].

The biosynthesis of polyphenols by plants is their response to environmental stressors (e.g., altitude, ultraviolet radiation, temperature, light conditions) [49,50,51]. Polyphenols can be found in the plant in the form of free molecules (which can produce one or more reactions) or bound molecules (responsible for transport and accumulation) [51]. Thus, Durdevic et al. [52] showed that in *Ceterach officinarum*, the content of free polyphenols (free phenolics) is higher than the content in bound polyphenols (bound phenolics). Based on the total polyphenol content, Lai and Lim [53] established different groups of ferns: *a*. ferns with very high TPC content (21–32 mg GAE/g f.w.), *b*. ferns with elevated TPC content (10–19 mg GAE/g f.w.), *c*. ferns with moderate TPC content (5–9.9 mg GAE/g f.w.), *d*. ferns with low TPC content (typically between 2.00 and 3.20 mg GAE/g f.w., respectively) [51]. The polyphenols content in ferns represent a very good indicator of a plausible elevated antioxidant potential of the fern extracts [54,55].

Although small, above-ground gametophytes, produce, in addition to chlorophyll pigments and carotenoids, polyphenols [56], ecdysteroids, anteridiogens, etc. For example, in *Polypodium vulgare*, the highest concentration of ecdysteroids is biosynthesized by the plant gametophyte [57]. Ecdysteroids may be used for phytophagous insect control, in gene-switch technology [58], for breast cancer treatment [59], etc.

Other constituents, like the hydrolysable and condensed tannins found in ferns, protect them from phytopathogenic insects [51]. The ferns contain a series of other promising biomolecules not explored to date for their application in nanotechnology. For example, sporopollenin, the main constituent of exospore and the perispore, is a mixed copolymer [60] with an aliphatic core, aromatic and phenolic groups, hydroxyls, ketones, and carboxylic acid esters [61]. The phytohormone abscisic acid (ABA) intervenes in this process of determining the sex of the gametophyte. The effect of ABA on the gametophyte is the opposite of that produced by the anteridiogen: ABA inhibits the development of the male phenotype. The spores contain large amounts of ABA, of the order of 40µM [62], which precipitate the day after germination. The decrease in ABA concentration causes the onset of the anteridiogen sensitive period. The ferns also contain various amounts of fatty acids and alkanes, pigments, carbohydrates, amino-acids, proteins and lipids, with lower concentrations of aldehydes, esters, ketones, and primary alcohols, resulting in a very complex and species-specific composition [63,64].

## 3. *Polypodiopsida* and the Nanomaterials

**Figure 2:**
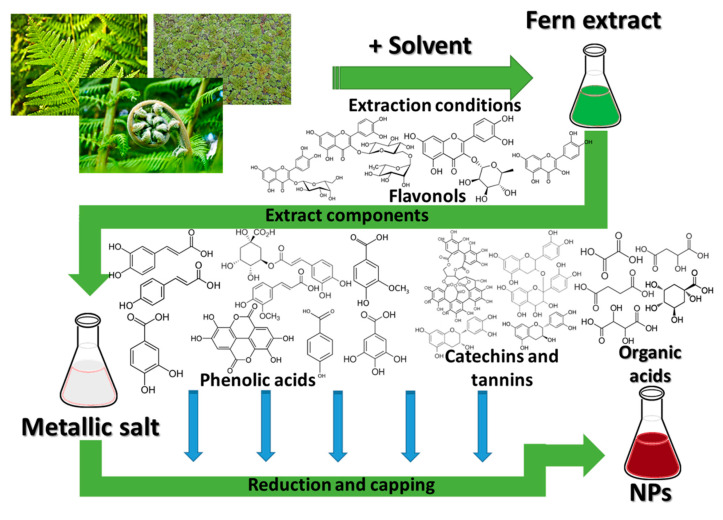
Phytosynthesis process and some of the involved phytoconstituents of ferns.

Although ferns are not as commonly encountered as other classes of plants in the nanotechnology area [18], the literature study revealed several important studies in this field, suggesting a potential field of application for this underutilized class, particularly in the nanomaterials phytosynthesis process. The phytosynthesis mechanism involves the reduction and stabilization of the metallic nanoparticles by the phytoconstituents of the vegetal material (Figure 2). As in the case of any phytosynthesis process, in the case of fern extracts, applications can be distinguished several factors influencing the morphology of the final nanoparticles (NPs) and thus their potential applications [18]:

- factors related to the vegetal extract used: the intrinsic properties of the plants, related to their phytocomponents, the part of the plant used, extraction procedure, used solvents, the vegetal material to solvent ratio, plant pre-treatment, etc.;
- factors related to the phytosynthesis process: concentration of the metallic salt precursors, reaction conditions (temperature, pH, reaction time), extract to metallic salt ratio, etc.

As all these factors can affect the NPs’ properties, studies should be considered for comparative evaluation of their influence. In the case of other plant classes, it is not surprising to identify studies on similar vegetal materials, with different results, as the authors used slightly different conditions; even the geographical region from which the vegetal material is collected can influence the NPs’ characteristics, as the plants’ composition can be influenced by the environmental factors [19].

The general process for the phytosynthesis of NPs using fern extracts, in which the main role for the reduction and capping of the metals is assigned to different phytoconstituents of the plants in the Polypodiopsida class, is depicted in Figure 2.

### 3.1. Nanoparticle Phytosynthesis Using Ferns

As the *terrestrial ferns* are the most encountered, their use for the nanoparticle phytosynthesis is also more frequent. Several authors present the phytosynthesis of different types of nanoparticles, most often silver or gold. Other types of NPs (copper oxide, iron) or composites are also encountered, although to a lesser extent. Among those nanoparticles, AgNPs are the most common subject of research, due to the well-known antimicrobial potential of silver, well-known from ancient times [65]. The differences between silver in the nanoparticle and in its ionic form, in terms of interaction with living cells, were recently discussed by other authors [65]. Elaborating the aspects detailed in the cited work, the use of silver in its nanoparticle form can be considered advantageous over the application of silver ions (even though some studies report inferior antibacterial properties for NPs, compared with silver ions [66]), due to several aspects:

- silver ions can bind to form different insoluble precipitates, which can negatively affect their properties [65];
- particularly for the case of phytosynthesized nanoparticles, the use of different phytocomponents as capping agents can not only contribute to an increase in their antimicrobial or antioxidant potential (for example) [18], but can decrease their toxic potential against non-target organisms [18], which is actually lower for NPs, compared with silver ions [67];
- the large surface area to volume ratio of nanoparticles (an element common for all types of NPs) provides better contact with microorganisms, thus increasing their antimicrobial potential, as well as contributing to their successful application in other areas [68].

Moreover, the tissue up-take of silver NPs and ions are rather similar, the effect being size-, shape-, dose- and capping agent- (for NPs) dependent [18,19,69]. These advantages are also applicable for other metallic nanoparticles, compared with their ionic form.

Of particular interest for the current review are the studies presenting the phytosynthesis of NPs achieved using different reaction parameters, for the same vegetal material. For example, the hart’s-tongue fern (*Asplenium scolopendrium* L.) was evaluated by our group for its ability to phytosynthesize silver nanoparticles. The change of the solvent and extraction method for the fern leaves extract led to the variation of the silver NPs’ characteristics, as well as to changes in their antimicrobial, cytotoxic and phytotoxic properties [17,47]. Additionally, the different parts used for the extraction (leaves or rhizomes) led to differences in the final properties of the NPs [47].

An edible terrestrial fern (*Diplazium esculentum* (Retz.) Sw.) was successfully evaluated by two groups [70,71] for the phytosynthesis of silver and silver and gold NPs, respectively, and for the study of their application in environmental protection. Their studies revealed the influence of the reaction temperature and of the ratio of extract to metallic salt on the formed metallic nanoparticles, as well as the differences recorded in terms of catalytic properties that are influenced by these changes in morphology. Miljković et al. [72] and Das et al. [73] used the leaves of field horsetail fern (*Equisetum arvense* L.) for the phytosynthesis of AgNPs with enhanced biomedical properties. Using the same plant parts (leaves) and solvent (water), under different extraction conditions (different vegetal material to solvent ration and different extraction method), the authors obtained similar morphologies (spherical, as most phytosynthesis processes for the AgNPs lead to spherical NPs), but very different sizes: 10–60 nm, respectively 170.5 nm (hydrodynamic diameter) [72,73]. Although the hydrodynamic diameter does not provide a “true” NP diameter, the UV-Vis spectrum presented by the authors (with an absorbance maximum at 488 nm) would suggest particles with diameters around 100 nm or beyond [74]. Miljković et al. [72] also studied the influence of other phytosynthesis parameters, such as pH (5–11), extract concentration (1–6, vol%) and temperature (20–100 °C), concluding that the optimum pH was 9, extract concentration 2% and lower temperature. The authors concluded that the acidic media hinders the phytosynthesis process (as a consequence of suppressed deprotonation of hydroxyl groups of polyphenols), while highly alkaline media (pH = 11) leads to the apparition of some larger aggregates.

Another fern that allows a similar discussion is the black maidenhair (*Adiantum philippense* L.), used by different authors to obtain AgNPs and AuNPs [75,76,77] with enhanced antimicrobial properties. Using the aerial parts of ferns and similar extraction methods and solvents, but different vegetal material to solvent ratios (1:20 and 1:50, respectively), Sant et al. [75] and Kalita et al. [76] obtained AuNPs with various shapes (mainly spherical and triangular) with different sizes: 10–18 nm (by TEM)/11 nm (by XRD), and 33.9 ± 14.0 nm, respectively (by TEM). By comparing the two studies, the most probable explanation for the differences in NPs dimensions is that the different vegetal material to solvent ratio led to a smaller polyphenolics content in the study of Kalita et al. [76], thus leading to larger particles. In the studies of Sant et al. [75] and Chatterjee et al. [77] regarding the application of the same fern for the phytosynthesis of AgNPs, the variation of the plant parts used for the extraction (aerial parts, respectively whole plant), using a similar extraction and phytosynthesis procedure, led to the formation of different sized NPs (13 nm and 28 nm, respectively, average diameters, by TEM), most probably due to the lower content of polyphenolics, under the influence of supplementary parts or due to environmental conditions.

Literature studies reviewed provides examples of the phytosynthesis of other types of nanoparticles, such as amorphous RuNPs (using *Nephrolepis biserrata* (Sw.) Schott leaves extract) with enhanced antimicrobial and antioxidant properties [78]. Iron-based nanoparticles (iron, iron oxide and FeOOH NPs) were obtained byYi et al. [79] using *Nephrolepis cordifolia* (L.) K. Presl and were evaluated for environmental applications (removal of Cr(VI)) [79]; crystalline CuONPs (6.5 ± 1.5 nm) were obtained by Sarkar et al. [80] using *Adiantum philippense* L. extract, suggesting their application as a potent plant defense booster (in experiments on *Lens culinaris* seeds).

Composite structures, such as Au–Ag@AgCl or SiO_2_@Au–Ag [81,82]. The phytosynthesis of Au–Ag@AgCl composites was achieved reducing the two metals with simultaneous generation of AgCl, the final composites with dimensions between 10 and 50 nm. The authors evaluated the application of the composite nanomaterial for the quinolines synthesis [81]. The same group obtained SiO_2_@Au–Ag composites using the same type of vegetal material (*Nephrolepis cordifolia* tuber extract). The SiO_2_ component of the composite, also phytosynthesized, had dimensions of 200–246 nm, while the noble metals NPs had average diameters of 3 nm. The composites’ catalytic, antibacterial and cytotoxic properties were successfully evaluated by the authors [82]. Further details on the phytosynthesis parameters and results obtained in terms of NPs characteristics are presented in Table 2.

Another widely spread category of ferns is represented by *aquatic ferns* (developing in aquatic ecosystems). Although their application in the phytosynthesis process is not as well developed as that for terrestrial ferns, several authors describe their application, mainly for the phytosynthesis of AgNPs or AuNPs. For example, four-leaf clover (*Marsilea quadrifolia* L.) leaf extracts were used for the synthesis of both AgNPs [83] and AuNPs [84,85], all with biomedical applications. Interestingly, by using different extraction conditions and solvent and vegetal material to solvent ratios, Chowdhury et al. [84] and Balashanmugam et al. [85] obtained very similar NPs in terms of shape (spherical) and size (17–40 nm and 10–40 nm, respectively). Most probably, by varying all these parameters, a similar composition was obtained for the extracts, which in turn led to similar morphologies for the NPs.

In general, most of the AgNPs phytosynthesized using fern extracts are spherical or quasi-spherical in shape (Table 2) (the most encountered morphology when speaking of phytosynthesized AgNPs [18]), while for AuNPs much diverse morphologies are encountered. For example, Abbasi et al. [86] obtained different sizes and shapes of AuNPs, by using different parts of the giant salvinia (*Salvinia molesta* D. Mitch.) aquatic fern (aerial/submerged parts) and different extract to metallic salt solution ratios. At lower extract/metal ratios smaller dimensions of nanoparticles for both types of plant parts were obtained (16.8–23.6 and 7–25 nm, respectively, by TEM), compared with the NPs obtained at higher ratios (20–50 nm and 75.5–175.8 nm, respectively, by TEM). Additionally, the lower-dimension NPs were mostly spherical, while for the conditions that led to higher dimensions NPs various morphologies were recorded (including triangular, pentagonal and nanoflower shapes).

A particular and rarely encountered approach (not only for the application of ferns) is represented by the study of Chumpol and Siri [87]. The authors present the in vivo formation of crystalline PbNPs, in the root cells of water velvet (*Azolla pinnata* R.Br.). Briefly, the method involved the uptake of lead ions in root cells of water velvet and their reduction to NPs. Additionally, the authors observed that the shape of the NPs was tissue-dependent, with spherical NPs in the epidermal cells, while short and long rod-shaped NPs were identified near the cell membrane of cortical and vascular cells, respectively. The authors assign the main role in the formation of NPs to the presence of strong reducing agents (i.e., quercetin) and weak reducing agents (i.e., tannic acid, glucose, etc.), while the main role as capping agents to the cellular complex molecules (i.e., carbohydrates, proteins, etc.), similar to that for the phytosynthesis of NPs using plant extracts.

Finally, two organs of *tree ferns* (*Cibotium barometz* (L.) J. Sm. and *Alsophila nilgirensis* (Holttum) R.M. Tryon) were also used for the phytosynthesis of metallic nanoparticles. Wang et al. [88] obtained spherical AuNPs (5–20 nm) and AgNPs (5–40 nm) using the water extract of woolly fern dried roots, while Johnson et al. [89] obtained spherical AgNPs using the sporophytes extract of the tree fern only growing on the Indian subcontinent *Alsophila nilgirensis* (Holttum) R.M. Tryon. Although rare, the application of tree ferns for the phytosynthesis process would suggest a potential use of other, wider-spread tree ferns.

Further details regarding the phytosynthesis of metallic nanoparticles using fern extracts are presented in Table 2.

**Table 2:** Some examples on the phytosynthesis of nanoparticles using ferns (presented alphabetically, by the family name; results obtained from the application studies are presented in Section 3.2.).^1.^

Fern	Family	Plant Part	Extraction Conditions	NP Characteristics	Intended Application	Ref.
***Terrestrial ferns***						
*Asplenium scolopendrium* L.	*Aspleniaceae*	Leaves, rhizomes	Ethanol, V.M.S.R. = 1:10, 48 h, R.T.	AgNPs, <50 nm	Antioxidant, cytotoxic	[47]
*Asplenium scolopendrium* L.	*Aspleniaceae*	Leaves	Water:ethanol, V.M.S.R. = 1:10, temperature extraction (2 h, 80 °C), microwave extraction (80 °C for 20 min., with magnetic stirring)	AgNPs, spherical, 12 nm (classical extraction), 10 nm (microwave extraction)	Antimicrobial, cytotoxic, phytotoxic	[17]
*Diplazium esculentum* (Retz.) Sw.	*Athyriaceae*	Leaves, powder	Dried powder directly applied in the metallic salt solution	AgNPs, spherical, oval, triangular, 10–45 nm	Photocatalytic, anticoagulative	[71]
*Diplazium esculentum* (Retz.) Sw.	*Athyriaceae*	Leaves, powder	Water, V.M.S.R. = 1:4.5, 70 °C, 20 min.	AgNPs, spherical, 10–25 nm (increased with extract concentration) AuNPs—spherical, triangular and decahedral shapes, 35–75 nm (dependent on reaction temperature and extract concentration)	Antimicrobial	[75]
*Pteridium aquilinum* (L.) Kuhn	*Dennstaedtiaceae*	Leaves	Water, V.M.S.R. = 1:10, boiling, 5 min.	AgNPs, spherical, 35–65 nm	Mosquitocidal, antiplasmodial	[90]
*Dryopteris crassirhizoma* Nakai (1920)	*Dryopteridaceae*	Rhizomes	Water, V.M.S.R. = 1:2.5, boiling, 30 min.	AgNPs, spherical, 5–60 nm (pH, extract, metallic salt and light exposure dependant)	Antibacterial	[91]
*Equisetum arvense* L.	*Equisetaceae*	Leaves	Water, V.M.S.R. = 1:25, microwave extraction (100 W for 3 min, 180 W for 1 min)	AgNPs, nearly spherical, 10–60 nm (phytosynthesis temperature dependant)	Antimicrobial	[72]
*Equisetum arvense* L.	*Equisetaceae*	Leaves	Water, V.M.S.R. = 1:4.58, boiled for 20 min under stirring.	AgNPs, spherical, hydrodynamic diameter, 170.5 nm	Cytotoxicity, antidiabetic, antioxidant, antibacterial	[73]
*Equisetum giganteum* L.	*Equisetaceae*	Leaves	Water, liquid-solid extraction, V.M.S.R. = 1:10, 80 °C, 3 min	AgNPs, spherical, 20 nm	Coating biodeterioration control	[92]
*Dicranopteris linearis* (Burm.f.) Underw	*Gleicheniaceae*	Leaves	Water, V.M.S.R. = 1:10, boiled for 5 min.	AgNPs, spherical, 40–60 nm	Mosquito oviposition deterrents	[93]
*Gleichenella pectinata* (Willd.) Ching	*Gleicheniaceae*	Leaves	Powdered sample soaked in methanol for 72 h at RT, with frequent manual agitation. V.M.S.R. = 1:10	AgNPs, spherical, 7.51 ± 2.88 nm.	Antimicrobial	[94]
*Nephrolepis biserrata* (Sw.) Schott	*Nephrolepidaceae*	Leaves	Methanol, V.M.S.R. = 1:8, 50 °C, 10 min.	RuNPs, hexagonal, amorphous, ~26 nm	Antifungal, antioxidant	[78]
*Nephrolepis cordifolia* (L.) K. Presl	*Nephrolepidaceae*	Tubers	Water, V.M.S.R. = 1:10, boiled for 15 min under stirring	Au–Ag@AgCl nanocomposites, spherical, 10–50 nm (average 30 nm)	Nanocatalysts	[81]
*Nephrolepis cordifolia* (L.) K. Presl	*Nephrolepidaceae*	Tubers	Water, V.M.S.R. = 1:20, boiled for 10 min	SiO_2_@Au–Ag nanocomposites, Spherical SiO_2_ decorated with AuNPs and AgNPs, 200–246 nm (SiO_2_), 3 nm (AgNPs/AuNPs)	Nanocatalysts, antibacterial, cytotoxic	[82]
*Nephrolepis cordifolia* (L.) K. Presl	*Nephrolepidaceae*	Not declared	Water, V.M.S.R. = 3:100, 80 °C for 80 min.	FeNPs, amorphous, spherical 40–70 nm, other iron oxides	Cr (VI) removal	[79]
*Adiantum philippense* L.	*Pteridaceae*	Aerial parts	Dried plant material, water, V.M.S.R. = 1:20	Anisotropic, AgNP, 13 nm, AuNP, 11 nm	Proposed for biomedical applications	[75]
*Adiantum philippense* L.	*Pteridaceae*	Aerial parts	Dried plant material, water, V.M.S.R. = 1:50, 25 °C for 72 h., under continuous shaking.	AuNPs, spherical and triangular, average particle size 33.9 ± 14.0 nm.	Antibacterial	[76]
*Adiantum philippense* L.	*Pteridaceae*	Whole plant	Dried plant material, water, V.M.S.R. = 1:20	AgNPs, quasi-spherical, 10–60 nm	Antimicrobial	[77]
*Adiantum philippense* L.	*Pteridaceae*	Whole plant	Dried plant material, water, V.M.S.R. = 1:20	CuONPs, quasi-spherical, 1–20 nm	Plant defence booster	[80]
*Pteris tripartita* Sw.	*Pteridaceae*	Leaves	Water, V.M.S.R. = 1:20, boiling for 5 min.	AgNPs, hexagonal, spherical, and rod-shaped, 32 nm	Anticancer, toxicity studies (on Zebra fish)	[95]
*Adiantum raddianum* C. Presl	*Pteridaceae*	Leaves	Dried leaf powder, water, V.M.S.R. = 1:10, 3 h., magnetic stirring	AgNPs, mostly spherical, with cubic morphologies, 9.69–13.9 nm	Mosquitocidal	[96]
*Adiantum capillus-veneris* L.	*Pteridaceae*	Leaves	Dried leaf powder, water, 70 °C, 15 min.	AgNPs, spherical, 18.4 nm	Antibacterial	[97]
*Adiantum capillus-veneris* L.	*Pteridaceae*	Leaves	Dried leaf powder, water, M.S.R. = 1:10, 60 °C, 10 min.	AuNPs	Antioxidant, antibacterial, antifungal	[98]
*Adiantum* sp.	*Pteridaceae*	Leaves	Aqueous extract	AgNPs, AuNPs	Anticancer	[99]
*Pteris quadriaurita* Retz.	*Pteridaceae*	Leaves	Dried leaf powder, water, M.S.R. = 1:10, 60 °C, 10 min.	AuNPs	Antioxidant, antibacterial, antifungal	[98]
***Aquatic ferns***						
*Marsilea quadrifolia* L.	*Marsileaceae*	Leaves	Water, V.M.S.R. = 1:20, 90 °C, 1 h., under stirring (400 rpm)	AgNPs, spherical, 9–42 nm	Antibacterial, anticancer	[83]
*Marsilea quadrifolia* L.	*Marsileaceae*	Leaves	Dried leaf powder, methanol, V.M.S.R. = 1:10, R.T., 72 h.	AuNPs, spherical, 17–40 nm	Antidiabetic	[84]
*Marsilea quadrifolia* L.	*Marsileaceae*	Leaves	Dried leaf powder, water, V.M.S.R. = 1:25, 55 °C, 15 min.	AuNPs, spherical, 10–40 nm	Antioxidant, cytotoxic	[85]
*Leptochilus pteropus* (Blume) Fraser-Jenk	*Polypodiaceae*	Leaves	Methanol, V.M.S.R. = ~1:6, 24 h., under magnetic stirring	AgNPs	Antioxidant	[100]
*Azolla pinnata* R.Br.	*Salviniaceae*	Whole plant	Dried powder, hydroalcoholic solution (70%/96%), percolation (48 h.), V.M.S.R. = 1:2.5	AgNPs, spherical, average size 6.5 nm	No application proposed	[101]
*Azolla pinnata* R.Br.	*Salviniaceae*	In vivo formation	Formation of NPs in root cells	PbNPs, spherical, rod-shaped, 12–80 nm dependent on the formation site	No application proposed	[87]
*Azolla filiculoides* Lam.	*Salviniaceae*	Whole plant	Dried powder, methanol, 72 h., V.M.S.R. = 1:10	AuNPs, spherical, 17–40 nm	Antioxidant, hepatoprotective	[102]
*Salvinia molesta* D. Mitch.	*Salviniaceae*	Whole plant	Dried plant, water, boiling, 5 min., V.M.S.R. = 1:100	AuNPs, various morphologies: spherical, triangular, pentagonal and nanoflower-like, dimensions ranging from 7 to 175.8 nm, dependent on the part plant used and extract to metallic salt ratio	No application proposed	[86]
*Salvinia molesta* D. Mitch.	*Salviniaceae*	Leaves	Water, boiling, 5 min., V.M.S.R. = 1:5	AgNPs, spherical, average size 12.46 nm	Antimicrobial	[103]
***Tree ferns***						
*Cibotium barometz* (L.) J. Sm.	*Cibotiaceae*	Roots	Dried powder, water, boiling, 30 min., V.M.S.R. = 1:20	AgNPs, spherical, 5–40 nm; AuNPs, spherical, 5–20 nm,	Antimicrobial, antioxidant, cytotoxic	[88]
*Alsophila nilgirensis* (Holttum) R.M. Tryon	*Cyatheaceae*	Sporophytes	Water, boiling, 30 min., V.M.S.R. = 1:10	AgNPs, spherical, 45–74 nm	Cytotoxic, phytotoxic	[89]

^1^ where: R.T.—room temperature, V.M.S.R.—vegetal material to solvent ratio.

### 3.2. Potential Applications of Phytosynthesized Nanoparticles

The phytosynthesis of the NPs leads to the attainment of nanoparticles with characteristics depending on the extract used. Being closely correlated with the natural extract, the phytosynthesized NPs finds applications in areas in which the extracts have a historical use, such as antioxidant or antimicrobial fields, in which various phytoconstituents (such as phenolic acids, flavonoids, terpenes, carotenoids and proanthocyanidins) have proven applicability.

The main area in which the fern-phytosynthesized NPs are expected to find application is represented by antimicrobial applications (and in this area are found most of the published studies on fern-mediated NPs). The antimicrobial mechanism of the phytosynthesized NPs is well established, being previously presented by our group [18], mainly involving the disruption of cellular membrane and on the generation of ROS (reactive oxygen species). Silver nanoparticles (a common subject of antimicrobial studies) represent the main subject regarding nanoparticles phytosynthesis using ferns. Multiple studies evaluated the antimicrobial potential of AgNPs obtained using fern extracts. Results (Table 3) presented either as inhibition zones or as MIC/MCBE (minimum inhibitory concentration/minimal concentration values for biofilm eradication) values are usually close to the positive control used for the experiments (a commercial antimicrobial). Significant results were obtained by Miljković et al. [72] using AgNPs obtained by *Equisetum arvense* L. especially against the Gram-negative bacteria, with an MIC of 0.72 mg/L.

AgNPs were proven as effective agents against several types of Gram-negative (*Pseudomonas aeruginosa*, *Escherichia coli*, *Aeromonas hydrophila*, *Klebsiella pneumoniae*, *Salmonella typhi*, *Salmonella enterica*, *Proteus vulgaris*, *Serratia marcescens*,* Vibrio cholerae*, *Shigella sonnei*, *Enterobacter aerogenes*) and Gram-positive bacteria (*Staphylococcus aureus*, *Bacillus cereus*, *Bacillus subtilis*, *Bacillus megaterium*, *Listeria monocytogenes*, *Enterococcus faecium*, *Listeria monocytogenes*) or fungi (*Candida albicans*, *Alternaria alternata*, *Chaetomium globosum*, *Aspergillus niger*, *Aspergillus flavus*, *Fusarium oxysporum*, *Penicillium chrysogenum*, *Rhizopus oryzae*). As previously discussed by our group [18,19], the main parameters that influence the antimicrobial properties of the nanoparticles are their size and shape. Particularly for the phytosynthesized NPs, the composition of the extract used for phytosynthesis also has a strong influence on the final antimicrobial properties, in some cases more important than the size of NPs [17]. As most of the AgNPs used for the antimicrobial assays had a similar spherical or quasi-spherical morphology, the size and capping phytoconstituents play the major role in the differences recorded between the antimicrobial activity of the different NPs. This aspect also represents one of the most important advantages of the phytosynthesized NPs: although by using other “green” methods, smaller dimensions can be achieved [104], the phytoconstituents usually enhance the antimicrobial properties of the silver NPs [17].

Other types of NPs, such as AuNPs show antimicrobial activity against a series of bacteria and fungi (*E. coli*, *P. aeruginosa*, *S. enterica*, *S. aureus*, *B. subtilis*, *Trichophyton rubrum*, *Scedosporium apiospermum*, *A. fumigates*, *A. niger*, *A. flavus*) with values of the inhibition zones comparable with the antibacterial/antifungal agent used as the positive control; again, the differences in effect could be assigned to the contribution of the phytoconstituents [98].

A rather exotic metallic nanoparticle (rarely encountered when discussing phytosynthesized NPs), RuNPs, showed antifungal activity in food poisoning techniques (against *Aspergillus flavus*), with a 50% inhibition at a 0.6-mL dose, compared with RuNPs phytosynthesized using other plants (sago palm, rosy periwinkle and holy basil), which presented a 50% inhibition at doses between 0.8 and 0.9 mL [78]. Although the NPs dimensions were higher compared with the other nanoparticles, the superior antifungal effect is assigned to the plant components (especially tannins and terpenoids). Larger composites (such as AuNPs–amoxicillin composites or SiO_2_@Au–Ag) [76,82] also presents antimicrobial activity against *E. coli*, *S. epidermis*, *B. subtilis*, *B. cereus*, *S. aureus*. In the study of Kalita et al. [61], where the AuNPs–amoxicillin was mainly proposed to reduce the cytotoxicity of the antibiotic, an enhanced bactericidal activity against Gram-positive and Gram-negative bacteria was observed, as well as a potent anti-MRSA (methicillin resistant Staphylococcus aureus) activity (increase in the inhibition zone from 56 to 90%, compared with the amoxicillin). The authors suggest a possibility subversion of antibiotic resistance mechanism by “overcoming the effect of high levels of β-lactamase produced by MRSA” [76]. The SiO_2_@Au–Ag composites exhibited (at a concentration of 200 mg/mL) an inhibition zone superior to the separated components (SiO_2_, AuNPs, AgNPs), very close to the values obtained for the positive control ampicillin (21 mm against *S. aureus*, and 14 mm against *E/coli*, compared with 23 and 18 mm, respectively, obtained for the positive control).

Phytosynthesized metallic nanoparticles further inhibit the cell cellular membrane and stop the mechanism behind synthesis of protein in the bacterial system. A higher concentration of NPs phytosynthesized possesses higher permeability than a lower concentration of NPs phytosynthesized and subsequently ruptures the cell wall of the microorganism [105]. Phytosynthesized metallic nanoparticles deactivate the microorganism and suppress their multiplication through interaction with microbial DNA, proteins, and enzymes [106].

The antimicrobial applications of the NPs phytosynthesized using fern extracts represent an important area of research, the results obtained being able to offer the instruments necessary for fighting health care associated and drug-resistant infections.

Multiple studies present the antioxidant properties of the phytosynthesized NPs, usually by comparison with the “parent” extract. The results of different in vitro assays reveal an enhancement of the antioxidant properties of the extracts upon the phytosynthesis. From the literature results surveyed, AgNPs present an IC_50_ (concentration required to result in a 50% antioxidant activity, compared with control sample) in the DPPH assay (the most used antioxidant assay) between 47 mg/L (small dimensions NPs) [100] and 92.9 mg/L (large NPs) [73]; AuNPs shows a much wider range of results (50 to over 1000 mg/L), while the only RuNPs study reports a value of 986 mg/L [78], a higher value, compared with the results obtained for the other NPs (which ranged from 389 to 692 mg/L). Results of other assays are presented in Table 3. Other authors present only the results only as DPPH radical inhibition, which makes the results harder to compare. However, as previously presented by our group [107], the in vitro antioxidant assays have little or no relevance for the living organisms, due to various characteristics of the involved assays. Those studies should be regarded only as preliminary evaluation results, who should be further confirmed by cell-lines experiments or in vivo assays.

Development of alternative agents with enhanced *cytotoxic potential* could offer next generation antitumoral instruments. Several plants were evidenced to possess cytotoxic potential [108], including ferns [109]. The evaluation of such natural alternatives even led to the development of commercially available, proven effective, antineoplastic chemotherapy drugs [110]. As such, this research area represents a particular interest, and the results, obtained either after in vitro preliminary studies or after more thorough in vivo studies, should motivate further investigations. The most encountered in vitro test for the evaluation of cytotoxicity is the *Allium cepa* assay. The chromosome aberration and micronucleus test can be successfully applied for the evaluation of different potential cytotoxic and genotoxic materials. Our group evaluated using this assay silver nanoparticles obtained using *A. scolopendrium* fern, identifying a progressive time-related mitoinhibitory effect, as well an increased frequency and variability of chromosomal aberrations for the phytosynthesized NPs, compared with the crude extracts [17,47]. Another in vitro test (MTT—3-(4,5-dimethylthiazol-2-yl)-2,5-diphenyltetrazolium bromide tetrazolium reduction colorimetric assay) is widely applied for the evaluation of detrimental effects on cellular metabolic activity. This test was also applied to determine the cytotoxicity of AgNPs and AuNPs and composites based on silver and gold NPs against a series of tumoral cell lines (Table 3), revealing a good cytotoxicity against the tumoral lines, in conjunction with a negative response on adipocyte cell lines, human embryonic kidney cells, human keratinocyte cells or normal subcutaneous areolar adipose tissue cellular lines (Table 3). For example, the composite SiO_2_@Au–Ag presented the highest cell viability (95% at 500 mg/L, 96 h), compared with the SiO_2_ (60% viability), SiO_2_@Ag (75%), SiO_2_@Au (approx. 90%). The higher viability was assigned by the authors to the binding of noble metals nanoparticles on the surface of the SiO_2_, causing their slow release. The results suggest the possibility of achieving a good selectivity of the developed NPs, or good compatibility, properties which can be harvested for the development of future anti-cancer drugs, respectively, for the development of biocompatible materials. Regarding the application of in vivo assays, spherical AgNPs were tested in the hatched shrimps bioassay, the authors obtaining an LC_50_ of 869.4 μL/10 mL, higher than with the used extract (1533.28 μL/10 mL), increasing in a dose-dependent manner (the presented results being obtained at a dose of 250 µL/50 mL) [89].

The larvicidal potential of AgNPs against known vectors of several viruses (*Anopheles stephensi, Aedes aegypti*, and *Culex quinquefasciatus*) is shown in Table 3. The results obtained in the laboratory assay can be compared regarding the LC_50_ recorded on the III instar larvae: on *Anopheles stephensi*, the lower dimension NPs showed an LC_50_ of 10.33 mg/L [96] compared with 13.77 mg/L, obtained for higher dimensions NPs [90]; on *Aedes aegypti*, the same lower dimensions NPs showed an LC_50_ of 11.23 mg/L [96], compared with 23.187 mg/L for higher dimensions [93]. The results (especially the results obtained in field larvicidal activity, by application in water storage tanks of concentrations ten times higher than the LC_50_) suggest their potential application for the reduction of populations of vectors mosquitoes of sever medical importance. More than that, the study of Govindarajan et al. [96] suggest their selectivity, as low toxicity being recorded against non-target organisms (such as the water bug—*Diplonychus indicus* Venk. et Rao or *Gambusia affinis* (S. F. Baird and Girard, 1853)—the western mosquitofish), with LC_50_ of 517.86, and 635.98 mg/L, against *D. indicus* and *G. affinis*, respectively [96].

The phytotoxic potential of phytosynthesized NPs can be harvested in developing next-generation, lower toxicity, targeted herbicides. The tests performed follow the effect of NPs on seeds of different model organisms (*T. aestivum, L. culinaris, V. radiata* or *S. vulgare*). The results suggested a reduction in seed germination present; however, the results presented by our group [17] suggested that, at optimum concentration, the NPs led to a reduction in the phytotoxic effect of the crude extracts. The evaluation of phytotoxicity of CuONPs using *Lens culinaris* seeds led to an increase up to 93.96/91.26 for NP concentrations of 0.01/0.025 mg/mL, compared with the control (80.77%), while at a higher concentration (0.05 mg/mL) the NPs became toxic, resulting in a decrease in seed germination (75.98%). The same tendency was also observed for seedling vigor index and relative water content. The authors concluded that, at a concentration of 0.025 mg/mL, CuONPs induced innate immunity and plant vigor. The higher tested concentration (0.05 mg/mL) were found to retard all the negative influence on the vegetal material, with increased stress markers, ROS, and H_2_O_2_ production [80].

Other important biological effects, scarcely presented by the literature, suggest the in vitro antidiabetic potential of AgNPs obtained using *Equisetum arvense* L. extract [73], the in vivo anti-inflammatory potential of AgNPs obtained using *Pteris tripartita* Sw. extract [95] and the hepatoprotective potential of the spherical AuNPs in the carp model [102].

Important catalytic properties were obtained for AgNPs in the degradation of dyes and organic compounds (methyl violet 6B, Rose Bengal, methylene blue, rhodamine B, 4-nitro phenol) [70,71]. More complex composites were evaluated for catalytic applications, such as Au–Ag@AgCl, successfully used in the synthesis of various quinoline derivatives, with a maximum yield of 96% and a recyclability of 88% after the fifth cycle [82], and SiO_2_@Au–Ag composites, applied for the solvent-free amidation of carboxylic acid, with a maximum yield of 97%, and a recyclability of 90% after the fifth cycle [81]. Phytosynthesized iron-based nanoparticles were also proven to be an efficient adsorbent for the removal of Cr(VI) (with a 90.93% removal) [79].

The results presented above (detailed in Table 3), are not intended to be an exhaustive presentation of all the potential applications of fern-phytosynthesized NPs, but a depiction of current interests in their application, which should be further enhanced in future studies.

### 3.3. Development of Biogenic Nanoparticles

Another nanotechnological-related potential application of the ferns is represented by the attainment of biogenic nanoparticles (especially silica). This application is related to the capacity of ferns to take up different metals or metalloids, followed by the processing of the vegetal material, in order to obtain the amorphous, semi- or highly crystalline nanoparticles. As such, the vegetal material can be considered a source of metalloids (in the case of silica). For example, Mattos et al. [111] obtained amorphous spherical SiO_2_ nanoparticles (7 nm) using horsetail fern (*E. arvense*) stems, by acid leaching (2% sulfuric acid, acid:solid ratio 10:1, temperature 100 °C), filtration and washing to neutral pH, drying (103 °C), and finally calcination for 1h at 650 °C in an air atmosphere. The authors used the biogenic silica as a carrier of a neem bark extract cross-linked with polycarboxylic acids biocide [112].

The same group used a three-step procedure to obtain agglomerated, irregular sphere-like silica nanoparticles from the leaves and stems of the same fern: hydroalcoholic extraction (1:1 (v/v) H_2_O: EtOH, solid:liquid ratio of 1:10, 24 h); hydrolysis of the pre-extracted biomass (diluted sulfuric acid or water, solid:liquid ratio 1:10, heated at different temperatures and times); calcination of the washed solid after the hydrolysis for 1 h. [113].

Hosseini Mohtasham and Gholizadeh [114] also used the horsetail (entire plant) to obtain semi-crystallin silica nanoparticles using a procedure involving acid leaching (4 M hydrochloric acid, acid:solid ratio 50:1, refluxed 2 h); the treated vegetal material was subsequently centrifuged and washed to neutral pH, dried (50 °C) and calcinated (air atmosphere, heating rate of 1 degree/min, 2 h at 500 °C). A final composite, consisting of H_3_PW_12_O_40_ loaded on the ethylenediamine immobilized on an epibromohydrin-functionalized Fe3O4@SiO_2_ support, was tested by the authors; magnetite deposited on the silica support was applied by the authors for the one-pot synthesis of dihydropyrano [2,3-c] pyrazole derivatives with a 99% yield [114].

Adinarayana et al. [115] used the water horsetail extract microwaved–pyrolyzed at 200 °C (30 min., using household microwave oven) for the attainment of highly crystalline silica nanoparticles (average size 2.5 nm) applied for the fluorescence detection of Fe^3+^ ions in water. Another species of the *Equisetum* genus (*Equisetum myriochaetum* Schltdl. and Cham., 1830—Mexican giant horsetail) was also used to obtain silica nanoparticles. Dried stems and branches were acid-digested (concentrated HNO_3_/H_2_SO_4_ = 4:1, solid:acid ratio = 1:40, 48 h.), washed to a pH of 5, lyophilized and calcinated (air atmosphere, 650 °C, 5 h, heating rate 10 °C/min) by Sola-Rabada et al. [116] to obtain amorphous, 5-nm biogenic silica, used for enzyme immobilization. Following the same recipe, Bogireddy et al. [117] obtained biogenic amorphous silica from the stems of the giant horsetail, functionalized it with APTES and grown PtNPs on the obtained substrate (using a chemical route). The composite was applied for the catalytic reduction of 4-nitrophenol to 4-aminophenol, obtaining a complete reduction in 90 s. at room temperature [117].

As can be seen from the above examples, some ferns were proven to have the ability to be used as important sources for the development of silica nanoparticles. This approach could be considered an important utilization of ferns, resulting in high added-value final composites.

## 4. Other Applications of Ferns in Nano and Biotechnology

### 4.1. Phytoremediation and Metal Up-Take

Soil contamination by heavy metals and metalloids is a huge problem which must be resolved, and phytoremediation is one of the solutions. Various plants have been identified that accumulate heavy metals, and most of them belong to the *Pteridophytes* family (*Pteris, Adiantum, Nephrolepis*, etc.). Ferns can be used in various ways in phytoremediation like: phytoextraction, phytodegradation, as hyperaccumulators, etc.

Hyperaccumulation of arsenic (As) was discovered in last few years and there is a small group of plants that are capable of isolating As in their above-ground structures at high concentrations; the majority arsenic-hyperaccumulators are ferns like: *Pteris vittata*, which has an extraordinary capacity to accumulate 2.3% arsenic in its biomass and *Pityrogramma calomelanos* which has the potential to remove approximately 2%. It has been confirmed that it survives in soil contaminated with copper (Cu), chromium (Cr), zinc (Zn) and, in addition, can effectively take up zinc into its leaf [118]. *Pteris vittata* is the best solution to use in phytoextraction, because it has a high biomass, it is a robust perennial and easy-to-grow plant, is resistant to disease and pests, and can tolerate 100 to 1000 times more arsenic than other plants. An explanation will be that *Pteris vittata* has a gene ACR3 that codes for the arsenous acid transfer protein [119].

A group of scientists from Singapore led by Dr. Lew [120] have accomplished a novel type of plant nanobionic optical sensor that can detect and monitor arsenic (in real time) in the belowground environment. Lew and co-workers have used a species of fern, *Pteris cretica*, which also can hyperaccumulate arsenic.

Pongthornpruek et al. [121] evaluated the accumulation of another heavy metal of some fern species. This research can play a huge role in the bioremediation process to minimize concentrations of heavy metals from the environment, as one of the tested ferns *Adiantum philippense* L. showed significantly higher levels of Ni and Pb concentration in their leaves, while *Adiantum caudatum* L. was the best at Pb, Ni and Co absorption.

Arsenic contamination of drinking water poses serious health risks to a huge number of people across the entire world. Removal efficiency of As for two arsenic-hyperaccumulating ferns (*P. vittata* and *P. cretica* cv. Mayii) and a non-accumulating fern (*Nephrolepis exaltata*) was examined by monitoring the depletion of ^73^As-labeled arsenic from the water [122]; the results from this research demonstrate that arsenic-hyperaccumulating fern species from the *Pteris* genus can rapidly remove arsenic from water. The high arsenic phytofiltration efficiency of *Pteris* ferns is related to their ability to translocate the absorbed As from roots to shoots; *Nephrolepis exaltata* does not have such a characteristic and is unable to reduce water arsenic concentrations.

In addition to drinking water, huge quantities of potentially toxic elements (PTEs), including dyes, drugs, cadmium, chromium, copper, mercury, lead, nickel, zinc, and arsenic have been released into aquatic ecosystems producing massive pollution.

Many aquatic ferns have a large potential for heavy metal removal from waters, especially in tropical and subtropical regions [123].

Given the above, Shafiqul Islam et al. [124] explored the phytotoxic properties of *Marsilea crenata,* an aquatic fern. An aqueous methanol extract showed inhibition on the seedling growth of cress, lettuce, alfalfa, barnyard grass, and foxtail fescue. The aqueous methanol extracts were purified by a chromatographic method and two phytotoxic substances were isolated and identified by spectroscopic analysis as loliolide and isololiolide; these compounds may be responsible for phytotoxic activity of this aquatic fern extract and could represent an important alternative for ecological agriculture.

Several aquatic ferns, such as *Azolla pinnata* and *Lemna minor* [125,126,127], have demonstrated the ability to accumulate PTEs from polluted water. These aquatic ferns can take up copper and decrease its concentration in the aquatic medium, and can be effectively used for the treatment of wastewater.

Zhang et al. [128] studied As accumulation of the aquatic fern *Azolla* and the results revealed that *Azolla caroliniana* accumulated two times more As than *Azolla filiculoides*. Compared to *Azolla* group, *Salvinia minima* Baker was capable to accumulate high concentrations of Pb, Ni and Cd but not As [129,130]. More than that, the ferns’ capacity to take up different metals and metalloids could allow, besides the phytoremediation of contaminated soils, the reclamation of rare elements from polluted sites [131].

### 4.2. Other Depollution Applications

As seen in the case of the *Salvinia* species, in addition to removing heavy metals, organic contaminants such as volatile compounds, oil, dyes, explosives, and hydrocarbons have been removed by fern species.

*Salvinia* can be used, in the form of dead biomass, to retain oil from oil/water emulsions, the results of the published study revealing an oil retention capacity of 1.33 g oil/g biomass, superior to the retention capacity of a commonly used oil sorbent (processed peat—0.26 g/g), the results attributed to a larger surface area and hydrophobicity [132]. *Salvinia* species, particularly *S. rotundifolia*, also have the capacity of treating groundwater contaminated with explosives, *S. rotundifolia* being found to convert explosives such as trinitrotoluene (TNT) to aminodinitrotoluene (ADNT, a metabolic product) [133].

*Azolla* species show biofiltration capacity as they can alter pH and remove chemical oxygen demand (COD), and polyphenols [134]. *A. pinnata* and *A. filiculoides* also possess the capacity to degrade hydrocarbon present in the growth medium [135,136].

Fern species, such as *Osmunda japonica, Davallia mariesii, Polypodium formosanum, Polypodium dispar, Polypodium multifida, Pteris spp.*, and *Pelargonium* spp., have shown efficacy for removing dyes, volatile organic compounds, such as formaldehyde [137] or even very toxic substances, such as hydrazine [138]. Biosorption of dyes by ferns is followed by their biotransformation, and detoxification into nontoxic metabolites [139]. Biotransformation is supported by intracellular enzymes such as tyrosinases and laccases. For instance, *Azolla filiculoides* removed the dye (e.g., C.I. Acid Blue 92) by an absorption process [140].

In addition to organic compounds and heavy metals, the ferns can also be used for the removal of other inorganic contaminants, such radionuclides; *Azolla caroliniana* have shown potential for removing several radioactive isotopes from radioactive wastewaters, such as ^137^Cs, ^60^Co, ^210^Po, ^109^Cd or ^238^U [141,142]. *Salvinia minima* possess capacity to remove inorganic nutrients such as ammonium and nitrate nitrogen [127]. Other potential contaminants, such as P, K, Mg and ammonia, were successfully removed from wastewater using *Azolla* species [143].

We can conclude that aquatic ferns have a high capacity to accumulate PTEs in their tissues and can be considered useful for the phytoremediation of residual water bodies contaminated with PTEs.

### 4.3. Phytotoxicity Assays

In addition to the previously mentioned properties, ferns are also used in phytotoxicity tests, although, according to the European Food Safety Authority [144], fern species are not used for regulatory purposes. Boutin et al. [145] classified the methods to achieve phytotoxicity tests and risk assessments and identified their certain related limitations, associated to the number of tested species and biodiversity; this is because most tests are centered on assessing the toxicity of herbicides to crops. Some studies [146,147,148] emphasize that ferns are quite sensitive to herbicides and thus could be applied for the phytotoxicity and ecotoxicity studies.

Rowntree and his team [146] investigated effects of the herbicide *Asulam* on eight fern species tested at maturity of sporophyte stage. Four of the studied species were affected by the high and medium doses of herbicides, and three of them were more sensitive than the flowering species *Rumex acetosa*. Similar studies, regarding the toxicity different pesticides were performed by Liu et al. [149] (evaluating the glyphosate effect on *Salvinia natans* (L.) All) and by De et al. [150] (evaluating the 2,4-Dichloro-phenoxy acetic acid effect on *A. pinnata* in the presence of TiO_2_ nanoparticles). Their conclusions, that TiO_2_ nanoparticles may alleviate the toxic effects, were also confirmed by Spanò et al. [151], who used *A. filiculoides* pre-treated with TiO_2_ NPs and evaluated the Cd-induced injuries in the model fern.

Catalá et al. [152] published the first bioassay of acute phytotoxicity based on fern spores; it is naturally miniaturized and combines biological and ecological relevance with sensitivity and simplicity. The salt used was TTC (2,3,5-triphenyltetrazolium chloride). The TTC method has been applied in the detection of fern spore viability of *Dryopteris guanchica*, from an environmental toxicity perspective. *Dryopteris guanchica* was chosen because the spores (fern spores are produced in large quantities) and leaves are produced throughout the year, and thus mature spores can be collected in different periods of time.

As a successful and inexpensive model for toxicity assays, the ferns were also applied to evaluate the toxic potential of different types of nanoparticles. Glenn et al. [153] evaluated the interactions of AuNPs with a series of aquatic vascular plants, including the fern *Azolla caroliniana* Willd. Unlike the other plants, the fern adsorbed both the small dimensions NPs (4 nm) and the larger dimension ones (18 nm), with no visual evidence of toxicity evidenced after 24 h. of exposure [153]. One the other hand, regarding the evaluation of ZnONPs’ effect on the fern *A. filiculoides,* Zarate-Cruz et al. [154] found a metallic oxide size-related effect, by comparison with the effect induced by the NPs (26.7 nm) with the ones of submicrometric particles (238 nm). The NPs reduced photosynthetic pigments, antioxidants, nitrogenase activity and chlorophyll fluorescence, suggesting a superior toxic effect of the nanoparticles, compared with the submicrometric particles [154]. A similar study was performed by Gómez-Garay et al. [155] regarding the side-related effect of CeO_2_ on *Asplenium adiantum-nigrum* spores. The results suggest an acceleration of the germination for the ceria nanoparticles (under 25 nm) at concentrations of 100–2000 mg/L, a similar effect being observed for the bulk ceria at 500–2000 mg/L. The toxicity of CeO_2_ against the fern manifested at cellular level through chloroplast membrane damage and lysis, disruption of the cell wall/membrane and morphological/developmental alterations. Nevertheless, the nanoparticles showed reduced toxicity at lower concentrations (100–500 mg/L), while the bulk ceria was toxic at all tested concentrations [155].

Species belonging to the *Azolla* genus (*A. filiculoides* and *A. caroliniana*) were used to evaluate the phytotoxicity of magnetite nanoparticles. If the phytosynthesized Fe_3_O_4_NPs (under 50 nm) negatively impacted development of *A. filiculoides* at concentrations between 0.5–10 mg/L (growth reduction, increased total phenols and flavonoid contents) [156], in atrazine-loaded magnetic poly(ε-caprolactone) microparticles (the magnetic phase being represented by 10.2 nm oleic acid coated superparamagnetic Fe_3_O_4_NPs), magnetite seemed to lower the toxicity of atrazine towards *A. caroliniana* [157].

The above-presented examples suggest a future potentially important application of the ferns in the nanotechnological area, respectively, for the evaluation of nanoparticles’ toxicity.

## 5. Conclusions

The species belonging to the *Polypodiopsida* class have been part of human culture since the beginning of civilization, often being utilized due to the presence of antimicrobial substances (such as alkaloids, terpenes as tannins, saponins, anthraquinones, cardiac glycosides, etc.) [158,159].

Extracts obtained from different parts of various fern species revealed important antioxidant and antibacterial activities [48], insecticidal [160,161], anti-inflammatory, analgesic [45,162], neuroameliorative properties [163] or even applications as an antiviral agent against SARS-CoV coronavirus [164]. The richness and variation of phytocomponents in the different fern species strongly supports their evaluation for different biomedical applications.

Considering the high number of research articles exploring the potential biomedical uses of ferns, numerous studies could be expected regarding their application in the nanotechnological area. As previously presented by our group, the nanomaterials phytosynthesis area represents a thriving research domain, with an exponential increase in the number of published papers seen in the last decade [18,19]. In this context, considering the known biomedical properties of ferns, as well as their widespread nature, the number of papers dealing with the phytosynthesis of nanomaterials via ferns is extremely low. This unexplainable bottleneck could be overcome by the orientation of various groups working in the “green” nanomaterials area towards this unexplored resource. This would, in turn, allow the comparison of the results between different works, as well as the optimization of the phytosynthesis process for the development of application-tuned nanomaterials (beyond the current state-of-the-art). At the same time, the variation of the metals or metal oxides envisaged by the phytosynthesis (i.e., Pd, Cu, SnO_2_, CeO_2_, ZrO, NiO, TiO_2_) or the synthesis of bimetallic structures would allow the enrichment of the final applications, as such leading to more in-depth knowledge regarding both the nanomaterials and the ferns. Additionally, the in vivo formation of nanoparticles using ferns represents a very interesting application, which takes us back to the first work regarding the nanoparticles’ phytosynthesis [165]. The tissue-specific morphologies and dimensions observed for the in vivo synthesis of nanoparticles would allow the easy development of nanoparticles with particular dimensions.

The metals and metalloids up-take capacity of ferns also allows different nanotechnological-related applications. On the one hand, this property could be an important instrument for the recovery of environmentally discharged NPs, while, on the other hand, this ability could be used for the attainment of different NPs, using further processing techniques of the fern biomass.

Not of secondary importance is also the potential use of ferns for the evaluation of NPs ecotoxicity. The inclusion of the ferns group in standard ecotoxicological tests is an important goal regarding ecological importance. Ferns, either aquatic (aquatic plants representing the main food producers in the aquatic ecosystems) or terrestrial (exploiting their relative developmental simplicity and speed), with their widespread nature and availability, can and should be considered as potential models for studying the toxicity of nanomaterials.

## Figures and Tables

**Table 1:** Synthetic classification of the *Polypodiopsida* class.

Class	Subclass	Order (Number)	Families (Number)	Genera (Number)	Species (Number)
*Polypodiopsida*	*Equisetidae*	1	1	1	15
	*Ophioglossidae*	2	2	12	129
	*Marattiidae*	1	1	6	111
	*Polypodiidae*	7	44	300	10,323
Extant ferns		11	48	319	10,578

**Table 3:** Potential applications of nanoparticles phytosynthesized using ferns ^1^.

Fern Used	Applied NPs	Application Results	Ref.
**Antimicrobial potential**			
*Asplenium scolopendrium* L.	AgNPs, 10–12 nm	Evaluated against *Staphylococcus aureus*, *Pseudomonas aeruginosa*; MIC-1/32 (against *S. aureus*); MCBE-1/16 (*P. aeruginosa*);	[17]
*Dryopteris crassirhizoma* Nakai (1920)	AgNPs, spherical, 5–60 nm	Evaluated against *Bacillus cereus* and *P. aeruginosa*; best inhibition zones (IZ): 10 mm/250 μg under green LED (*B. cereus*); 6 mm/250 μg under green LED (*P. aeruginosa*).	[91]
*Equisetum arvense* L.	AgNPs, nearly spherical, 10–60 nm	Evaluated against *Escherichia coli*, *S. aureus*, *Candida albicans*, commercial probiotic *Saccharomyces boulardii*; Selective activity against *E. coli* (effective at low concentrations-0.72 mg/L);	[72]
*Equisetum arvense* L.	AgNPs, spherical, 170.5 nm	evaluated against *Salmonella enterica, B. cereus, Listeria monocytogenes, Enterococcus faecium, S. aureus, Aeromonas hydrophila*) IZ (mm) = 11.64/10.75/12.46/9.68/12.53/10.80	[73]
*Equisetum giganteum* L.	AgNPs, spherical, 20 nm	Evaluated against *E. coli, S. aureus,* *Alternaria alternata, Chaetomium globosum*; Fungal resistance test and antibacterial biofilm tests after incorporation in waterborne paints; active against all strains; MIC-3.3/13.3/3.3/67.5 μg/mL; paint films inhibited fungal and bacterial biofilm development	[92]
*Dicranopteris linearis* (Burm.f.) Underw	AgNPs, spherical, 40–60 nm	Evaluated against *Bacillus subtilis, Klebsiella pneumoniae* and *Salmonella typhi*; IZ (mm) = 21.01/20.1/19 at 75 ppm	[93]
*Gleichenella pectinata* (Willd.) Ching	AgNPs, spherical, 7.51 nm.	Evaluated against *P. aeruginosa, E. coli, K. pneumoniae* and *C. albicans*; IZ (mm) = 15/11/10/13 at 5 mM	[94]
*Nephrolepis biserrata* (Sw.) Schott	RuNPs, ~26 nm	Evaluated against *Aspergillus flavus*; 50% inhibition at 0.6 mL;	[78]
*Nephrolepis cordifolia* (L.) K. Presl	SiO_2_@Au–Ag composites (200–246 nm SiO_2_ decorated with 3-nm AuNPs/AgNPs)	Evaluated against *E. coli, S. aureus*; IZ (mm) = 21/14	[82]
*Adiantum philippense* L.	AuNPs—spherical, triangular, 33.9 nm in AuNPs–amoxicillin composites	Evaluated against *E. coli, S. aureus, Staphylococcus epidermis, B. subtilis, B. cereus*, MRSA1, MRSA2, MRSA3, MRSA4; in vivo treatment of systemic MRSA infection : IZ (mm) = 31/30/19/35/38/16/15/12/12 MIC/MBC (mg/L) = 2/4; ½; ½; 16/32; 8/16; 16/32; 16/32; 16/32; 32/32. Survival rate at day 7 post-inoculation 96%	[76]
*Adiantum philippense* L.	AgNPs, quasi-spherical, 10–60 nm	Evaluated against *B. subtilis*, *Listeria monocytogenes*, *S. aureus*, *E. coli*, *K. pneumoniae, Salmonella typhimurium*; MIC = 105.41/17.55/17.85/12.36/17.84/28.77	[77]
*Pteris ripartite* Sw.	AgNPs, different morphologies, 32 nm	Evaluated against *B. subtilis*, *B. cereus, Bacillus megaterium*, *E. coli*, *Proteus vulgaris*, *Serratia marcescens*, *S. typhi*, *K. pneumoniae*, *Vibrio cholerae*, *Shigella sonnei*, *Enterobacter aerogenes*, *P. aeruginosa, A. niger*, *Aspergillus flavus*, *Fusarium oxysporum*, *Penicillium chrysogenum*, *Rhizopus oryzae*; IZ (mm) = 8.33 (*B. cereus*) − 24.33 (*P. aeruginosa*) at 10 mg/mL; MIC (at 10 mg/mL, 24 h) = 0.29 (*P. aeruginosa*) − 1.40 (*E. coli*);	[95]
*Adiantum capillus-veneris* L.	AgNPs, spherical, 18.4 nm	Evaluated against *E. coli* and *S. aureus*; IZ (mm) = 30/19 applied as “thick nanoparticle suspension”	[97]
*Adiantum capillus-veneris* L.	AuNPs	Evaluated against *E. coli, P. aeruginosa, Salmonella enterica, S. aureus, B. subtilis, Trichophyton rubrum, Scedosporium apiospermum, Aspergillus fumigates, A. niger, A. flavus*; IZ (mm) = 16 (*A. fumigates, S. apiospermum, S. enterica*) − 21 (*E. coli*)	[98]
*Pteris quadriaurita* Retz.	AuNPs	Evaluated against *E. coli, P. aeruginosa, S. enterica, S. aureus, B. subtilis, T. rubrum, S. apiospermum, A. fumigates, A. niger, A. flavus*; IZ (mm) = 14 (*T. rubrum*) − 18 (*S. aureus*)	[98]
*Marsilea quadrifolia* L.	AgNPs, spherical, 9–42 nm	Evaluated against *E. coli*; MIC = 0.5 nM;	[83]
*Salvinia molesta* D. Mitch.	AgNPs, spherical, 12.46 nm	Evaluated against *E. coli, S. aureus*; IZ (mm) = 21/16 (at 50 ppm); MIC = 10.5/13 mg/L Cell viability loss = 95.8/92.6% after 8 h. at MIC	[103]
*Cibotium barometz* (L.) J. Sm.	AgNPs, spherical, 5–40 nm;	Evaluated against *E. coli, S. aureus, S. enterica, P. aeruginosa*; IZ (mm, AgNPs) = 16/17.5/12.5/12.5 at 45 μg/disc;	[88]
**Antioxidant potential**			
*Asplenium scolopendrium* L.	AgNPs, < 50 nm	DPPH inhibition 81.34%/80.93% (rhizomes/leaves mediated NPs)	[47]
*Equisetum arvense* L.	AgNPs, spherical, 170.5 nm	IC_0.50_ (reducing power activity) = 641.24 μg/mL; IC_50_ (ABTS/DPPH/NOx) = 210.16/92.90/62.52 μg/mL;	[73]
*Nephrolepis biserrata* (Sw.) Schott	RuNPs, ~26 nm	IC_50_ (mg/mL, DPPH, ABTS, SORS, HSA assays) = 0.986/0.852/1.265/1.389	[78]
*Pteris tripartite* Sw.	AgNPs, different morphologies, 32 nm	DPPH, chelating activity, Phosphomolybdenum, ABTS, HPSA assays: 47.90 (mg/L)/61.51 ± 0.61 (mg EDTA/g)/41.94 ± 2.29 (mg AAE/g)/8592.70 ± 614.2 (μmol Trolox/g)/16.20 ± 3.86 (%);	[95]
*Adiantum capillus-veneris* L.	AuNPs	Inhibition: ~90% (DPPH)/~70% (SORS)/~85% (HPSA)/~82% (HSA);	[98]
*Pteris quadriaurita* Retz.	AuNPs	Inhibition: ~81% (DPPH)/~60% (SORS)/~77% (HPSA)/~75% (HSA);	[98]
*Marsilea quadrifolia* L.	AuNPs, spherical, 10–40 nm	IC_50_ (DPPH) = 50 mg/L;	[85]
*Leptochilus pteropus* (Blume) Fraser-Jenk	AgNPs	IC₅₀ = 47.0 μg/mL (DPPH)/35.8 μg/mL (HPSA)	[100]
*Cibotium barometz* (L.) J. Sm.	AgNPs, spherical, 5–40 nm; AuNPs, spherical, 5–20 nm,	IC_50_ (DPPH) = 1.4/1.22 mg/mL (AuNPs/AgNPs)	[88]
**Cytotoxic potential**			
*Asplenium scolopendrium* L.	AgNPs, < 50 nm	Rhizomes extract mediated NPs-progressive time-related mitoinhibitory effect; for both NPs—increased frequency and variability of chromosomal aberrations in the *Allium cepa* assay	[47]
*Asplenium scolopendrium* L.	AgNPs, 10–12 nm	Significantly higher frequency of the total aberrant cells compared with the negative control sample in the *Allium cepa* assay	[17]
*Equisetum arvense* L.	AgNPs, nearly spherical, 10–60 nm	MTT assay (MC3T3-E1): Cytotoxic threshold: >2.25/> 4.5 mg L^−1^, lower for smaller NPs	[72]
*Equisetum arvense* L.	AgNPs spherical, 170.5 nm	Trypan blue exclusion test (HepG2): 20% viability (at 1 mg/mL)	[73]
*Nephrolepis cordifolia* (L.) K. Presl	SiO_2_@Au–Ag composites (200–246 nm SiO_2_ decorated with 3 nm AuNPs/AgNPs)	MTT assay (human keratinocyte cells): 95% cell viability at 500 μg/mL	[82]
*Adiantum philippense* L.	AuNPs-spherical, triangular, 33.9 nm.	MTT assay (L929): 81% viability (AuNPs), 79% viability (AuNPs–amoxicillin composites)	[76]
*Adiantum* sp.	AgNPs, AuNPs	MTT assay: Cytotoxicity against MCF-7 cells at different concentrations (2.5 to 100 μg/mL); non-cytotoxic to HEK293 cells	[99]
*Marsilea quadrifolia* L.	AgNPs, spherical, 9–42 nm	MTT assay: Cell death: 40.04% (MCF-7)/55.88% (HeLa), with NP sonication	[83]
*Marsilea quadrifolia* L.	AuNPs, spherical, 17–40 nm	MTT assay (3T3-L1): Cell viability = 71.23% (100 μM) − 84.02% (30μM); glucose uptake = 60.86%	[84]
*Marsilea quadrifolia* L.	AuNPs, spherical, 10–40 nm	MTT assay: IC_50_ = 45.88/52.01 mg/L (PA-1/A549)	[85]
*Cibotium barometz* (L.) J. Sm.	AgNPs, spherical, 5–40 nm; AuNPs, spherical, 5–20 nm,	MTT assay (RAW264.7 and MCF-7): AuNPs—no cell death at 0.1–10 mg/L; AgNPs—cytotoxicity at ≥ 10 mg/L against RAW264.7	[88]
*Alsophila nilgirensis* (Holttum) R.M. Tryon	AgNPs, spherical, 45–74 nm	Hatched shrimps bioassay: LC_50_ = 869.4 μL/10 mL	[89]
**Larvicidal potential**			
*Pteridium aquilinum* (L.) Kuhn	AgNPs spherical, 35–65 nm	Against *Anopheles stephensi* Liston, 1901 in laboratory conditions: LC_50_ of 7.48 ppm (larva I), 10.68 ppm (II), 13.77 ppm (III), 18.45 ppm (IV), and 31.51 ppm (pupae); Larvicidal assays in the field: complete removal of *An. stephensi* population after 72 h (at 10 × LC_50_ in water reservoir)	[90]
*Dicranopteris linearis* (Burm.f.) Underw	AgNPs spherical, 40–60 nm	Against *Aedes aegypti* (Linnaeus in Hasselquist, 1762); laboratory conditions: LC_50_ = 18.905 ppm (I)/ 20.929 ppm (II)/23.187 ppm (III)/26.312 ppm (IV)/29.328 ppm (pupae); LC_90_ = 32.140 ppm (I)/35.489 ppm (II)/39.696 ppm (III)/44.418 ppm (IV)/48.511 ppm (pupae) Field larvicidal activity (by application in water storage tanks), ovicidal assay, oviposition deterrent activity 100% reduction in A. aegypti larval populations at 10 × LC_50_ (after 72 h); No hatching observed at 25 ppm; ER = 94.29% at 30 ppm;	[93]
*Adiantum raddianum* C. Presl	AgNPs, 9.69–13.9 nm	Against mosquito larvae, in laboratory conditions (*A. stephensi, A. aegypti*, and *Culex quinquefasciatus* Say, 1823): LC_50_ = 10.33/11.23/12.19 mg/L Low toxicity against non-target organisms (*Diplonychus indicus* Venk. et Rao and *Gambusia affinis* (S. F. Baird and Girard, 1853)), LC_50_ = 517.86–35.98 mg/L	[96]
***Phytotoxic potential***			
*Asplenium scolopendrium* L.	AgNPs, 10–12 nm	NPs led to the reduction of the phytotoxic effect of the extracts in Triticum test	[17]
*Adiantum philippense* L.	CuONPs, quasi-spherical, 1–20 nm	Effect on *Lens culinaris* Medik: 91.26% seed germination, SVI = 4168.43, RWC = 84.37% at 0.025 mg/mL (optimum dose); optimum dose showed highest activity of defense enzymes and total phenolics; higher concentrations of NPs retard all the parameters	[80]
*Alsophila nilgirensis* (Holttum) R.M. Tryon	AgNPs, spherical, 45–74 nm	Effect on *Vigna radiata* (L.) R. Wilczek and *Sorghum vulgare* (L.). Seeds: Germination reduction: 38.65/100% (*V. radiata* and *S. vulgare*) at 50 mg/L	[89]
**Antidiabetic potential**			
*Equisetum arvense* L.	AgNPs, spherical, 170.5 nm	IC_50_ (alpha-glucosidase) = 1.73 μg/mL;	[73]
**Anti-inflammatory potential**			
*Pteris tripartite* Sw.	AgNPs, different morphologies, 32 nm	Anti-inflammatory activity Wistar albino adult female rats using the carrageenan-induced paw oedema method = 56.36%, 24 h., 100 mg/kg b.w.	[95]
**Hepatoprotective potential**			
*Azolla filiculoides* Lam.	AuNPs, spherical, 17–40 nm	Significant increase in cell viability compared to the acetaminophen group (hepatocytes damage); significant reduction in the levels of LDH and CAT (dose dependent); AuNPs significantly reduced the GOT and GPT levels (50/10%), significantly increased the levels of GSH-Px and SOD (60–70%), drastically reduced the formation of MDA (60%) and ROS generation	[102]
**Catalytic properties**			
*Diplazium esculentum* (Retz.) Sw.	AgNPs, different morphologies, 10–45 nm	Degradation of MB and RhB dyes under solar light illumination: complete disappearance of the adsorption peaks after 8 min.	[71]
*Diplazium esculentum* (Retz.) Sw.	AgNPs—spherical, 10–25 nm; AuNPs-different morphologies, 35–75 nm	Degradation of MV 6B, RB, 4-nitro phenol: Ag-98.4/98/96.8%; Au-98.2/98.9/97.3%	[70]
*Nephrolepis cordifolia* (L.) K. Presl	Au–Ag@AgCl, average size 30 nm	Synthesis of quinoline derivatives via three component coupling/hydroarylation/dehydrogenation of arylaldehyde, aniline, and phenyl acetylene derivatives; 96% yield for the composite applied, reaction conditions 9h, at reflux	[81]
*Nephrolepis cordifolia* (L.) K. Presl	SiO_2_@Au–Ag composites (200–246 nm SiO_2_ decorated with 3 nm AuNPs/AgNPs)	Solvent-free amidation of carboxylic acid catalyst: 97% yield for the composite applied, reaction conditions—8 h, 100 °C	[82]
**Other environmental applications**			
*Nephrolepis cordifolia* (L.) K. Presl	FeNPs, spherical, 40–70 nm, other types of iron oxides	Cr(VI) removal: 90.93%	[79]

^1^ where: 3T3-L1—adipocyte cell lines; A549—adenocarcinomic human alveolar basal epithelial cells line; AAE—ascorbic acid equivalents; ABTS—azinobis 3-ethylbenzothiazoline-6-sulfonate; b.w.—body weight; CAT—catalase; DPPH—2,2-diphenyl-1-picrylhydrazyl; EDTA—ethylenediaminetetraacetic acid; ER—effective repellence; GOT—glutamate oxalate transaminase; GPT—glutamate pyruvate transaminase; GSH-Px—glutathione peroxidase; HEK293—human embryonic kidney 293 cells; HeLa—human cervical cancer cell line; HepG2—human liver cancer cell line; HPSA—hydrogen peroxide scavenging activity; IC_50_—concentration required to result in a 50% antioxidant activity; LC_50_—LC_50_ lethal concentration that kills 50% of the exposed organisms; LDH—lactate dehydrogenase; HSA—OH^–^ scavenging activity; L929—normal subcutaneous areolar adipose tissue cellular lines; MB—methylene blue; MBC—minimum bactericidal concentration; MC3T3-E1—mouse pre-osteoblast cells; MCBE—minimal concentration values for biofilm eradication; MCF-7—breast cancer cell line; MDA—malondialdehyde; MI—mitotic index; MIC—minimum inhibitory concentration; MP—mitotic phases; MRSA—Methicillin-resistant *Staphylococcus aureus;* M.V. 6B—Methyl Violet 6B; NOx—nitric oxide; PA-1—human ovarian teratocarcinoma cell line; RAW264.7—macrophage, Abelson murine leukaemia virus transformed cells line; RB—Rose Bengal; RhB—rhodamine B; ROS—reactive oxygen species; RWC—relative water content; SOD—superoxide dismutase; SORS—Superoxide Radical Scavenging; SVI—Seedling Vigour Index.
